# Initial stability and stress distribution of ankle arthroscopic arthrodesis with three kinds of 2-screw configuration fixation: a finite element analysis

**DOI:** 10.1186/s13018-018-0972-1

**Published:** 2018-10-20

**Authors:** Min Zhu, Cheng-song Yuan, Zhong-min Jin, Yun-jiao Wang, You-xing Shi, Zhi-jin Yang, Kanglai Tang

**Affiliations:** 1 Department of Orthopaedic Surgery, Southwest Hospital, The Third Military Medical University, Gaotanyan Str. 30, Chongqing city, 400038 People’s Republic of China; 2 School of Mechanical Engineering, Southwest Jiaotong University, Chengdu, 610031 Sichuan China

**Keywords:** Arthrodesis, Arthritis, Ankle, Arthroscopy, Finite element analysis

## Abstract

**Background:**

Arthroscopic ankle arthrodesis (AAA) is recognized as the standard treatment for the end-stage ankle arthritis. Two-screw configuration fixation is a typical technique for AAA; however, no consensus has been reached on how to select most suitable inserted position and direction. For better joint reduction, we developed a new configuration (2 home run-screw configuration: 2 screws are inserted from the lateral-posterior and medial-posterior malleolus into the talar neck) and investigated whether it turned out to be better than the other commonly used 2-screw configurations.

**Methods:**

In this study, we investigated three kinds of 2-screw configurations: 2 “home run”-screw configuration (group A), crossed transverse configuration (the screw is inserted from the medial malleolus into the anterior talus and the other from the lateral tibia maintains posterior talus, group B), and 2 parallel screw configuration (2 parallel screws are inserted from the posteromedial side of the tibia into talus, group C). The effects of the above three insertions on the loading stress of the tibio-talar joint were comparatively analyzed with a three-dimensional finite element model.

**Results:**

Group A was better than groups B and C in respect of stress distribution uniformity and superior to both groups B and C in anti-flexion strength and anti-internal rotation strength. Group A was slightly worse than group C but better than group B in anti-dorsiflexion and anti-valgus and varus strength.

**Conclusions:**

Two “home run”-screw configuration facilitates the reduction of anterior talus dislocation of end-stage ankle arthritis. Our finite element analysis demonstrates the configuration is superior to crossed transverse and parallel configuration for arthroscopic ankle arthrodesis in terms of stress distribution and initial stability.

## Background

Ankle arthrodesis is still the most important treatment for the end-stage ankle arthritis [1]. Recently, arthroscopic ankle arthrodesis (AAA) has been shown to offer the advantages of better clinical scores, fewer complications, a shorter hospital stay, and less blood loss compared with open ankle arthrodesis (OAA) [2, 3]. Cannulated screws are commonly utilized for internal fixation. Guide pins can be inserted to check the radiographic position before the final screw fixation. Various double-screw configurations are used for internal fixation [4–8]. The post traumatic arthritis mostly occurs in ankle [9, 10], and the talus usually moves forward or dislocation at end-stage. Therefore, we inserted one “home run”-screw form posterior malleolus to anterior into the talus neck pulling the talus back and fixed [11–13]. This was referred as a new 2 home run-screw configuration and analyzed for the corresponding initial stability and stress distribution compared with the other two methods by the finite element analysis to examine the superiority.

## Methods

### Establishment of three-dimensional (3D) model

A healthy male volunteer was selected who was 37 years old and had a height of 175 cm and a body weight of 70 kg; foot tumor, deformation, and other lesions were excluded. The ankle scan was obtained from weight-bearing computed tomography (WBCT) using the PedCAT unit (Curvebeam, Warrington, USA). Triangular mesh models of the tibia, and talus were reconstructed using a visualization software package (Mimics 19.0 Materialize, Leuven, Belgium). The triangular meshed model was imported into a reverse engineering software (Geomagic studio 10.0, Geomagic, Research Triangle Park, NC) and translated triangle mesh surface into NURBS surface and exported as *.iges file. The file was imported into a three-dimensional CAD software (ProE Wildfire), where the talus and tibia components were assembled according to the original anatomical location. There were no contacts between the initial position of the talus and the tibia because of the lack of cartilage in the model. So, the talus was moved up by 1.5 mm as compensation. The cancellous screws were simplified as 6.5-mm-diameter cylinders in ProE Wildfire; and the thread was not modeled, and instead, a contact interface was used to take its effect into consideration.

### Experimental grouping and finite element analysis

#### 3D modeling

**Fig. 1:**
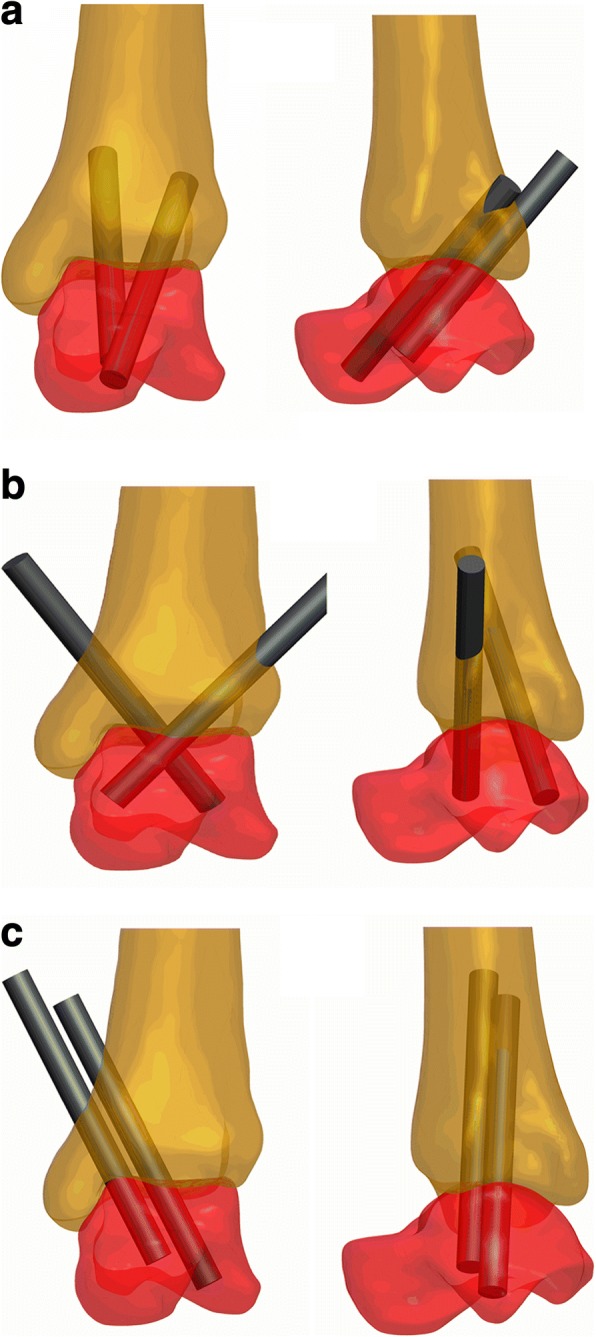
Three kinds of 2-screw configuration of ankle arthrodesis, displayed in frontal and lateral views (**a** group A, **b** group B, **c** group C)

Screw model and the fused tibia-talar joint model were assembled according to three kinds of 2-screw configurations for AAA. Three different forms of assembly were used to represent the 2 “home run”-screw configuration (2 screws are inserted from the lateral-posterior and medial-posterior malleolus into the talar neck, group A, Fig. 1a), crossed transverse configuration (the screw is inserted from the medial malleolus into the anterior talus and the other from the lateral tibia maintains posterior talus, group B, Fig. 1b), and 2 parallel screws configuration (2 parallel screws are inserted from the posteromedial side of the tibia into talus, group C, Fig. 1c). The three models were exported in x_t * format and imported in the finite element software (ABAQUS 6.13,Dassault Systems Simulia Corp., Providence, RI, USA). Since only the tibia near the fixation was important in this analysis, a 75-mm length was taken at the distal end of the tibia for later analysis. Tibia and talus bones were considered as linear elastic materials. Because of the elastic modulus of screws were much larger than that of the bone and in order to improve the computational efficiency of the model, the screws were set as rigid bodies. The corresponding material parameters are listed in Table 1.

**Table 1:** Material parameters

	Material	Elastic modulus (MPa)	Poisson’s ratio
Talus	Elastic	13,000	0.3
Tibia	Elastic	8370	0.3
Screw	rigid body	110,000	0.3

#### Loads and boundary conditions for the screw fixation

**Fig. 2:**
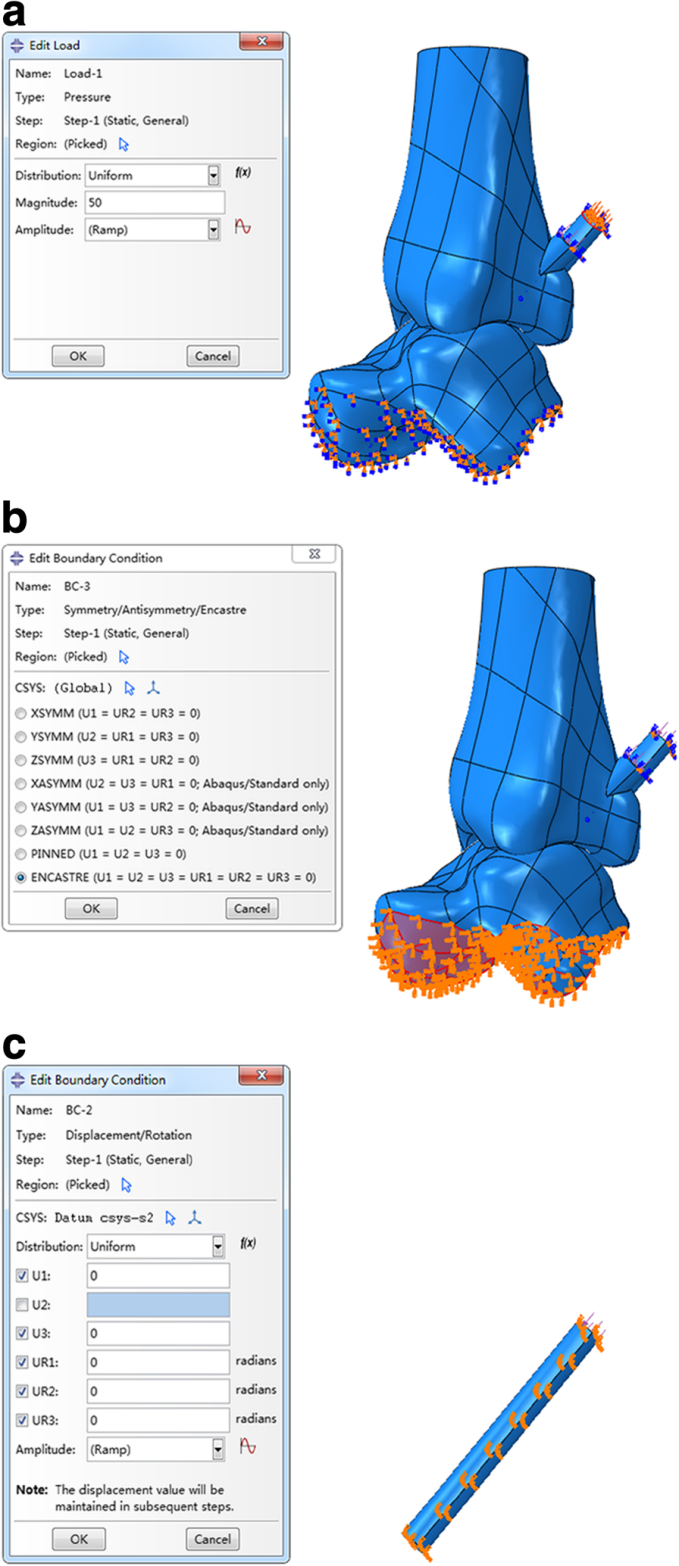
**a** 50 MPa perpendicular to each screw top surface. **b** The six degrees of freedom on the lower surface of the talus. **c** The screws were restrained to move along their axes

A local coordinate system was created, with the origin chosen at the center point of the screw. In the local coordinate system, the Y direction was the axial direction of the screw, and the X and Z directions were two mutually perpendicular radial directions of the screw section. Both load and boundary conditions were created in the local coordinate system. A uniform pressure distribution of 50 MPa perpendicular to each screw top surface was applied to simulate the external force (Fig. 2a). The 6° of freedom on the lower surface of the talus was constrained (Fig. 2b), and the screws were restrained to move along their axes (Fig. 2c).

#### Loads and boundary conditions for gait

After the implantation, the load and boundary conditions were reserved. A moment of 10 Nm was applied to the upper surface of the tibia to simulate the load of varus, valgus, flexion, dorsiflexion, external rotation, and internal rotation respectively (Tables 2, 3, and 4).

**Table 2:** Spatial displacement at nodes on tibia contact surfaces (μm)

Configuration	Flexion	Dorsiflexion	Valgus	Varus	External rotation	Internal rotation
Group A	28.44	24.83	28.33	29.06	31.01	32.41
Group B	29.30	34.12	31.53	33.46	24.00	42.80
Group C	32.84	21.70	25.08	24.68	21.35	48.23

**Table 3:** Rotational displacement at the tibia upper surface in the direction of loading (micro-radians)

Configuration	Flexion	Dorsiflexion	Valgus	Varus	External rotation	Internal rotation
Group A	2.238	2.045	1.905	2.184	2.721	3.609
Group B	2.449	2.946	2.076	2.264	1.228	4.101
Group C	3.046	1.707	1.420	2.063	2.897	4.245

**Table 4:** Stresses at nodes on tibia contact surfaces (maximum/average MPa)

Configuration	Flexion	Dorsiflexion	Valgus	Varus	External rotation	Internal rotation
Group A	64.97/2.477	70.63/2.568	70.97/2.676	66.45/2.535	61.00/2.756	84.24/2.759
Group B	49.03/2.714	46.66/3.066	51.15/2.894	50.13/2.913	40.57/2.568	60.52/3.479
Group C	90.56/2.745	83.50/3.022	79.44/2.901	90.47/2.924	91.29/3.358	88.97/3.091

#### Interaction

Various contacting components were considered as interacting surfaces, including between talus-tibia, screw-talus, and screw-tibia. A contact pair was defined for each potential interacting surfaces, with the considerations in the normal direction with the default setting (hard contacts), and tangential direction with a friction coefficient. Furthermore, “finite sliding” was considered to model potential nonlinear effects. The details of all the contact pairs are shown in Table 5.

**Table 5:** Interaction settings in the FE model

Contact pair	Interaction		
	Normal behavior	Tangential behavior	Sliding formulation
Talus-tibia	“Hard” contact	Coef. of friction friction μk = 0.1	Finite sliding
Screw-talus	“Hard” contact	Frictionless	Finite sliding
Screw-tibia	Tie		
Screw	Rigid body		

#### Mesh

Due to the irregularity of the bone surface, tetrahedral mesh was used for the talus and tibia (C3D4: 4-node linear tetrahedron element), and hexagonal mesh for the screws (C3D8R: 8-node linear brick, reduced integration, hourglass control element). In order to improve the efficiency and accuracy of the finite element analysis, the mesh of contact surface of tibia and talus were refined. Group A was meshed a larger and a smaller number of nodes additionally for mesh sensitivity analyses. Both the maximum von Mises stress of the two models were differed from the standard model by < 3%.

## Results

### Stress distribution of ankle arthrodesis

**Fig. 3:**
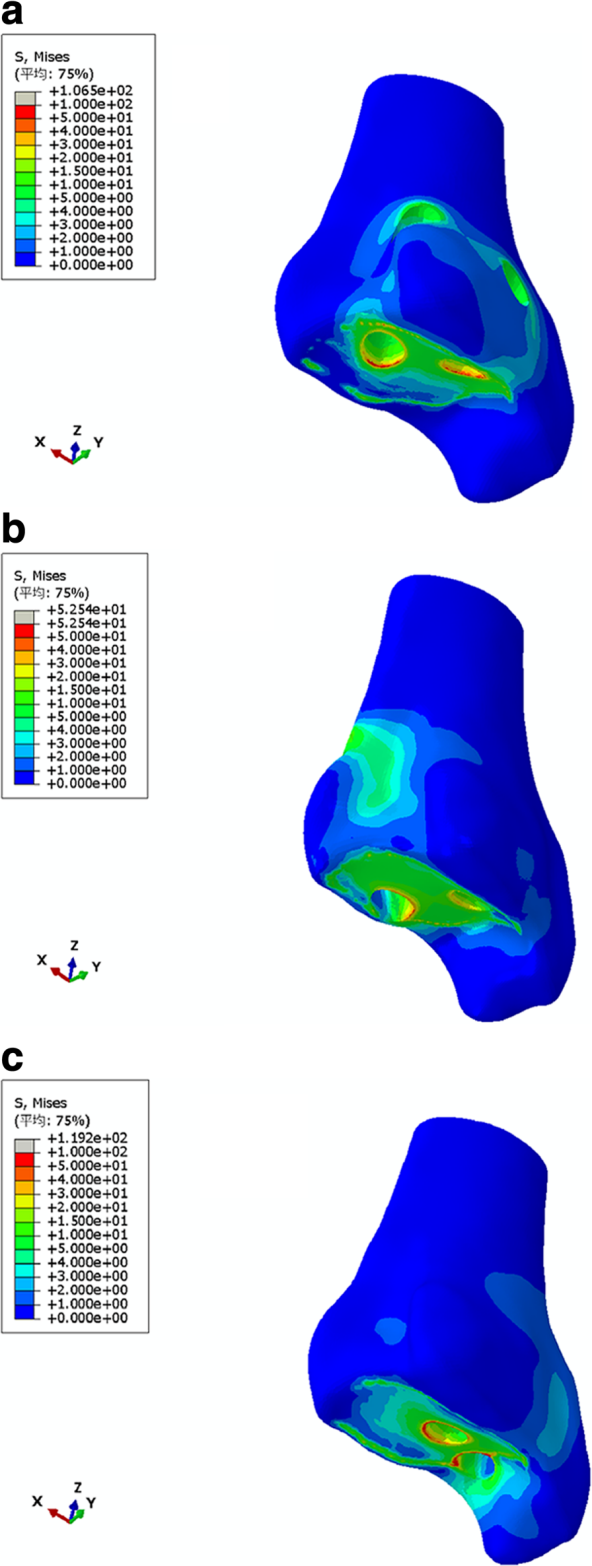
Von Mises stress distribution of tibia (**a** group A, **b** group B, **c** group C)

**Fig. 4:**
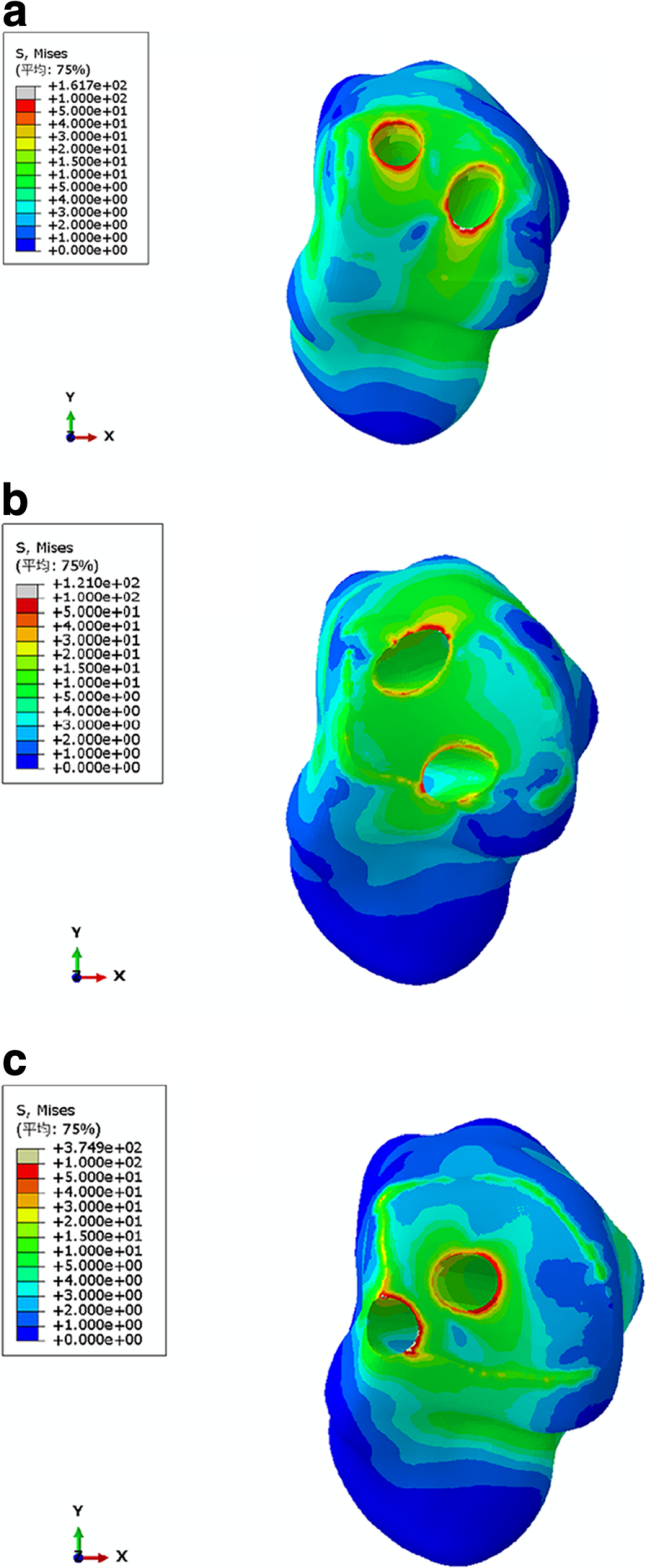
Von Mises stress distribution of talus (**a** group A, **b** group B, **c** group C)

**Fig. 5:**
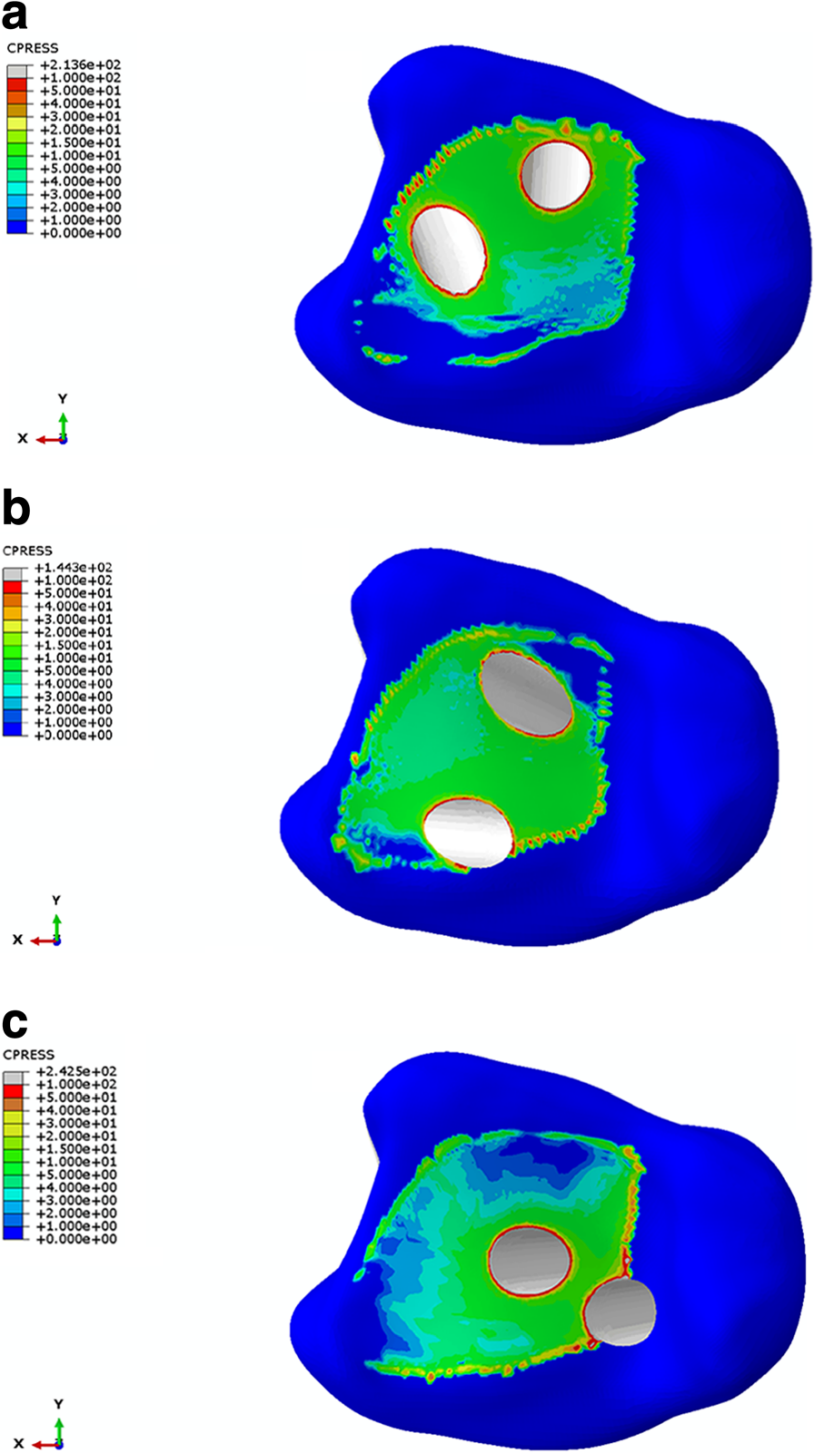
Contact pressure at tibia contact surface nodes (**a** group A, **b** group B, **c** group C)

**Fig. 6:**
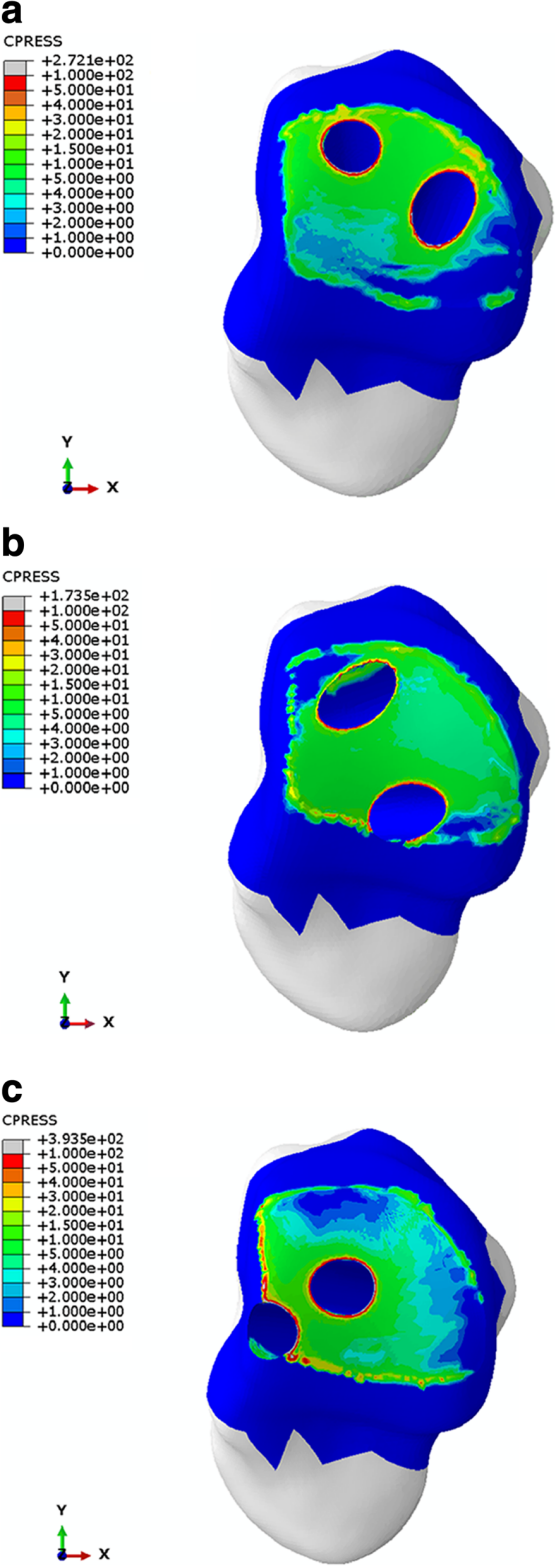
Contact pressure at talus contact surface nodes (**a** group A, **b** group B, **c** group C)

Considering that the models contained many nodes and elements, the maximum stress of each part, the maximum, and the average von Mises stress of the contact surface were calculated and are shown in Table 6 and Figs. 3 and 4. The contact pressure at the tibia-talus contact surface nodes were calculated and shown in Table 7 and Figs. 5 and 6. The contact areas of the articular surface of each model are shown in Table 8.

**Table 6:** Stress distribution of three different ankle arthrodesis (MPa)

Configuration	Part	Maximum von Mises stress	Maximum von Mises stress on contact surface	Average von Mises stress on contact surface
Group A	Tibia	106.49	67.27	2.40
	Talus	161.67	146.45	7.69
Group B	Tibia	52.54	45.05	2.75
	Talus	121.01	121.01	5.75
Group C	Tibia	119.20	82.01	2.29
	Talus	374.86	284.94	4.83

**Table 7:** Contact pressure at tibia-talus contact surface nodes (MPa)

Configuration	Part	Maximum contact pressure	Average contact pressure
Group A	Tibia	213.63	9.18
	Talus	272.09	7.06
Group B	Tibia	144.32	8.92
	Talus	173.45	6.78
Group C	Tibia	242.53	9.03
	Talus	393.46	6.83

**Table 8:** Contact area of articular surface (mm^2^)

Configuration	Contact area
Group A	325.19
Group B	342.00
Group C	341.15

### Initial stability of ankle arthrodesis

It is generally assumed that a more stable fixation leads to a higher chance of fusion, so the micromotions at the articular surface, as well as the structural strength of the implant (screw and bone), were focused in this analysis. Because the numbers of the nodes on the fracture contact surfaces of the four models were different, the average absolute displacement U and von Mises stress of the tibia contact surface and displacement of the tibia upper surface (UR) were taken as the evaluation parameters of the initial stability of ankle arthrodesis (Tables 2 and 3).

## Discussion

AAA offers fusion rates of greater than 90%, with low complication rates and a shorter time to union. Two cannulated percutaneous screw fixation has commonly been used for stabilization of AAA, and there are many configurations available for delivery of the screws across the tibio-talar interface. The orientation of the screws was still important in determining the stability of a two-screw configuration [14]. Crossed or parallel transmedial and/or translateral malleolar screws were used for internal fixation for arthrodesis [8, 15–17]. Most articles regarding AAA have recommended preparation of the tibial and fibular articular surfaces and the insertion of crossed transmalleolar screws. However, Ichiro et al. [18] observed loosening in 75% of cases with transmalleolar crossed screw fixation, without union in the talofibular joint. They suggest that there is no requirement to fuse the talofibular aspect in cases without severe osteoarthritis of the talofibular joint, and it is recommended that an additional screw should be inserted in the transtibial direction.

The talus usually moves forward or dislocation at the end-stage ankle arthritis [9, 10]. There is anatomical predisposition of anterior translation of the ankle—the anterior tilt of the distal tibia. The normal sagittal plane joint line orientation of the ankle has been described as the anterior tilt of the distal tibia or anterior inclination of tibia [19]. In Magerkurth’s studies [20], the values were 83 ± 3.6°. Obtaining ideal position and rigid fixation for fusion has the absolute priority, while understanding reduction and compression is critical to ankle arthrodesis. A screw from the posterior surface of the tibia into the talar head and neck has been called the “home run screw,” which allows maximum purchase into the body of the talus and serves to firmly secure the talus in a posterior attitude [12]. In the previous methods, the intraoperative reduction was poorly maintained, and the postoperative fusion time was also uneven [18].

In the recent years, the finite element analysis method has been widely applied in the research of orthopedic biomechanics. In analyzing the internal stress distribution and strain state of bones and the stress distribution of internal fixation and quantitatively analyzing the stress concentration and stress shielding, it has achieved the study results difficultly acquired by object reality experiments, as compared with the ordinary mechanical experiment methods; thus, it is of important clinical significance [21]. Our study confirmed our hypothesis in part. We developed 2 “home run”-screw configuration and turned out to be better than the other commonly used 2-screw configurations.

Although many different biomechanical studies for AAA techniques have been performed, we are not aware of any study comparing new double-screw system with the classic two-screw fixation technique. There are several limitations for our study. First, our sample sizes with only one volunteer’s hindfoot CT. However, the data show that despite the differences in the cause of different volunteer or patient, the affected only is a single amount of data, and the overall trend does not change. Second, the experimental setup does not represent physiologic gait. Nevertheless, dorsiflexion is the major stress for a fused ankle during normal gait [22]. Our double “home run”-screw configuration stress is distributed on the back side of the articular surface, which may be more conducive to ankle fusion after whole weight bearing.

## Conclusions

Two “home run”-screw configuration facilitates the reduction of anterior talus dislocation of end-stage ankle arthritis. The present FEA demonstrates the configuration has a greater primary stability and stress distribution when compared with crossed transverse and parallel configuration. Whether this will improve postoperative rehabilitation after ankle fusion or not is still subject for future clinical and radiographic research.

## Abbreviations

3D: Three-dimensional
AAA: Arthroscopic ankle arthrodesis
FEA: Finite element analysis
OAA: Open ankle arthrodesis
